# EBF1 Deficiency Drives Prostate Cancer Progression by Interfering with the Transcriptional Regulation of *ITPR1*

**DOI:** 10.32604/or.2026.078850

**Published:** 2026-06-16

**Authors:** Yuan Guo, Hui Wang, Xiaoyu Zhao, Xiao Feng, Xingjian Cai, Wei Li, Xiaohui Luo

**Affiliations:** 1 Department of Urology, Xi’an International Medical Center Hospital, Xi’an, China; 2 Department of Basic Psychology Teaching and Research, Military Medical Psychology Section, Fourth Military Medical University, Xi’an, China; 3 Department of Human Anatomy, Histology and Embryology, Basic Medical Science Academy, Fourth Military Medical University, Xi’an, China; 4 Department of Urology, Baoji Center Hospital, Baoji, China

**Keywords:** Prostate cancer, early B-cell factor 1 (EBF1), transcriptional regulation, inositol 1,4,5-trisphosphate receptor type 1 (ITPR1), tumor suppressor gene

## Abstract

**Backgrounds:** Early B-cell factor 1 (EBF1), originally identified as a critical transcription factor modulating the development and differentiation of B lymphocytes, has recently attracted significant interest owing to its diverse functional characteristics and regulatory mechanisms in solid tumors. Although current evidence suggests a potential connection between EBF1 and oncogenic developments, its exact role in the progression of prostate cancer (PCa) is unclear. This study sought to investigate its biological roles and regulatory mechanisms in human PCa. **Methods:** Bioinformatic analyses were performed utilizing Tumor Immune Estimation Resource (TIMER) 2.0, Gene Expression Profiling Interactive Analysis (GEPIA), and Gene Expression Omnibus (GEO) databases, along with quantitative polymerase chain reaction (qPCR) assays and immunostaining on clinically available PCa and normal prostate tissues, to investigate the expression profile and clinical significance of EBF1. The manipulation of EBF1 expression through lentivirus was conducted to explore the impact of both knockdown and overexpression of EBF1 on the malignant behaviors of PCa cells *in vitro*, as well as in a xenograft mouse model. Ultimately, various molecular biomedical techniques were employed to clarify the possible transcriptional regulation of inositol 1,4,5-trisphosphate receptor type 1 (ITPR1) by EBF1. **Results:** Bioinformatic analyses revealed that reduced *EBF1* expression exhibited a robust prognostic association with PCa progression. From a functional standpoint, knockdown of EBF1 promoted cell proliferation (n = 3, *p < 0.05, ***p < 0.001 for LNCaP-48 h and LNCaP-72 h; *p < 0.05, ****p < 0.0001 for 22RV1-48 h and 22RV1-72 h respectively), colony formation (n = 3, ***p < 0.001 for LNCaP and **p < 0.01 for 22RV1), migration (n = 3, ***p < 0.001 for LNCaP and ***p < 0.001 for 22RV1) and invasion (n = 5, ****p < 0.0001 for LNCaP and ****p < 0.0001 for 22RV1) capabilities in LNCaP and 22RV1 cells, while ectopic overexpression of EBF1 in PC-3 cells significantly inhibited tumor growth (n = 5, *p < 0.05 at d 36 after cell inoculation) and invasiveness (n = 5, **p < 0.01 for E-cadherin and Vimentin H-scores, respectively) *in vivo*. Mechanistically, EBF1 depletion was associated with a reduced expression of ITPR1, a key player in intracellular calcium release and a fundamental tumor suppressor in multiple cancers. Furthermore, using chromatin immunoprecipitation (ChIP), electrophoretic mobility shift (EMSA) and luciferase reporter assays, we demonstrated that EBF1 could directly bind to the distal region of the *ITPR1* promoter (n = 3, **p < 0.01 for ChIP-qPCR), thereafter facilitating its transcriptional expression (n = 3, ****p < 0.0001 for reporter assays). Importantly, overexpression of exogenous ITPR1 effectively ameliorated EBF1 deficiency-induced tumorigenicity in both LNCaP and 22RV1 cells. **Conclusions:** These findings together indicate a new function of EBF1 loss in enhancing PCa progression, highlight ITPR1 as a key downstream effector in facilitating the tumor-suppressing function of EBF1, and provide significant insights into improving our understanding of the transcriptional regulation of malignant behaviors in PCa cells.

## 1 Introduction

Prostate cancer (PCa) has a substantial global impact, representing the second leading cause of cancer-related mortality in men worldwide [1]. In 2020, it ranked as the 6th and 7th most common cancer by incidence and mortality, respectively, among Chinese males [2]. In contrast to other forms of cancer, PCa frequently displays a slow-growing indolent nature characterized by an extended clinical trajectory, which can, in certain instances, last over 15 years after the initial diagnosis. This indolent progression creates a therapeutic window for early intervention, potentially modifying the disease trajectory before aggressive transformation [3]. Given that the growth of prostatic tumors, whether benign or malignant, is highly influenced by the male hormonal environment, modulation of testosterone activity, via suppression of secretion or inhibition of androgen receptor (AR) interaction, is a standard treatment approach for metastatic PCa. Although androgen deprivation therapy (ADT) effectively controls hormone-naïve PCa, a subset of malignant cells evade dependence on androgen signaling through pathway alterations, culminating in castration-resistant prostate cancer (CRPC), a lethal and treatment-refractory subtype [4]. The poor outcomes of these lethal castration-resistant PCa variants highlight the urgent need for the development of new therapeutic strategies.

The progression of tumors is linked to the upregulation or downregulation of essential genes and particular pathways that promote cell division. Consequently, these genes and pathways are primary targets for developing novel anti-cancer medications [5]. Early B-cell factor 1 (EBF1), a transcription factor critical for B-lymphocyte development and differentiation, is implicated in the pathogenesis, relapse, and therapeutic resistance of B-cell acute lymphoblastic leukemia (B-ALL). The primary role of EBF1 is to aid in metabolic regulation, remodeling of the tumor microenvironment, genetic variation, and epigenetic modification throughout the progression of the disease [6]. Nevertheless, accumulated evidence indicates that EBF1 is a versatile regulatory factor that demonstrates a variety of functions and regulatory mechanisms that vary across different tumor types and their microenvironments [7]. EBF1 has been closely associated with both physiological and pathological processes, including adipocyte differentiation [8], bone metabolism [9], and inner ear development [10]. Additionally, EBF1 inactivation may cause dysregulation over these developmental pathways, ultimately leading to tumorigenesis. In this regard, EBF1 inactivation has been observed in various cancers, including brain tumors, liver carcinoma, colon cancer, breast cancer, and head and neck squamous cell carcinoma [7]. Notably, in PCa cells, overexpression of long intergenic noncoding RNA 00844 (LINC00844) can decrease proliferation and promote apoptosis by augmenting the EBF1 signaling pathway [11]. Similarly, protein-protein interaction (PPI) network analysis has identified the regulatory network of nuclear receptor subfamily 2 group F member 2 (NR2F2) and its potential target genes (EFNB2, EBF1, ETS1, and VEGFA) as the hub genes related to PCa progression [12]. However, these studies have limitations: (i) neither included clinical samples to establish EBF1’s prognostic relevance in PCa, and (ii) neither elucidated the downstream mechanisms mediating EBF1’s effects.

We hypothesize that EBF1 acts as a tumor suppressor by maintaining transcriptional homeostasis in prostate cells. This study aims to (i) profile EBF1 expression in PCa versus normal prostate tissues and (ii) define its clinical and mechanistic relevance in disease progression. Furthermore, this study aims to explore the effects and molecular mechanisms associated with the manipulation of EBF1 expression on the malignant phenotypes of PCa cells, using multiple molecular biological techniques. Overall, our systematic analysis is expected to broaden the understanding of EBF1’s role in PCa.

## 2 Materials and Methods

### 2.1 Bioinformatics Methodology

We utilized the Tumor Immune Estimation Resource (TIMER) 2.0 portal (https://compbio.cn/timer2/) to explore the expression levels of EBF1 across various cancer types (pan-cancer). TIMER2.0 employs raw RNA-Seq data from The Cancer Genome Atlas (TCGA) and provides normalized expression data in Transcripts Per Million (TPM) format. To analyze the differential expression of EBF1 between PCa tumor tissues and adjacent normal tissues using TIMER2.0, the following parameters were specified: Targeted gene (EBF1).

We also employed the Gene Expression Profiling Interactive Analysis (GEPIA) database (http://gepia.cancer-pku.cn/) to investigate EBF1 expression in PCa progression. GEPIA analyzes RNA sequencing expression data based on TCGA and GTEx projects. To assess differential EBF1 expression between PCa tumors and normal tissues using GEPIA, the following parameters were set for the Expression DIY module:

· Dataset: PRAD (Prostate adenocarcinoma);
· Gene: EBF1;
· Use GTEx data? Yes (to utilize GTEx normal tissue data);
· |log2FC| Cutoff: 1;
· *p*-value Cutoff: 0.05 (Note: The specific statistical test used by GEPIA, typically Wilcoxon rank-sum test, is implied by the parameter selection but not explicitly stated in the interface description).

To perform survival analysis on PCa patients stratified by EBF1 expression levels using GEPIA’s Survival Analysis module, the following parameters were configured:

· Method: Overall Survival (OS) or Disease-Free Survival (DFS) (as applicable per analysis);
· Group Cutoff: Quartile;
· Hazard Ratio (HR): Yes;
· 95% Confidence Interval (CI): Yes;
· Axis Units: Months;
· Datasets Selection: PRAD.

To evaluate the correlation between EBF1 and ITPR1 (Inositol 1,4,5-Trisphosphate Receptor Type 1) mRNA expression levels within PRAD tumor samples using GEPIA’s Correlation Analysis module, the following parameters were entered:

· Gene A: EBF1;
· Gene B: ITPR1;
· Correlation Coefficient: Spearman;
· TCGA Tumor Dataset: PRAD;
· TCGA Normal Dataset: PRAD Normal.

Additionally, we analyzed gene expression microarray datasets from the Gene Expression Omnibus (GEO) database (https://www.ncbi.nlm.nih.gov/geo/) using the GEO2R tool for differential EBF1 expression:

Dataset GSE3325: Comparison of PCa tumor tissues versus benign prostate tissues.

Dataset GSE34839: Comparison of spontaneous prostate tumors derived from Pten-null mice versus normal mouse prostate tissue. (Note: This dataset pertains to a mouse model, distinct from human TCGA/GTEx data).

### 2.2 Clinical Samples and Ethics Statement

The experimental protocols that included human specimens in this research adhered to the *Declaration of Helsinki* and received approval from the Medical Ethics Board of Baoji Center Hospital (Approval No. BZYL2023-20). Following the collection of signed informed consents from each participant, we proceeded to enroll 37 cases of fresh benign prostatic tissues from patients who had undergone radical cystoprostatectomy as a result of bladder cancer, along with 91 cases of hormone naïve clinically localized PCa tissues from patients undergoing radical prostatectomy (without previous hormonal deprivation therapy) from May 2023 to June 2025 at the Department of Urology in Baoji Center Hospital. The clinical features of the patients enrolled in this study are summarized in Supplementary Table S1. This research was conducted in accordance with the ethical principles outlined in the Declaration of Helsinki. The obtained biopsies were either immersed in 10% buffered formalin at room temperature (RT) for a duration of 6 to 8 h for paraffin embedding, or they were snap-frozen in liquid nitrogen and later preserved at −80°C.

### 2.3 Cell Treatment

Human prostate cancer cell lines (LNCaP, 22RV1, and PC-3) along with the normal human prostate cell line RWPE-1 were sourced from the American Type Culture Collection (Genetimes ExCell Technology, Shanghai, China). All cell lines were confirmed to be mycoplasma-free by polymerase chain reaction (PCR) testing and authenticated by short tandem repeat (STR) profiling before experimental use. RWPE-1 cells were grown in a keratinocyte serum-free medium (Thermo Fisher, Shanghai, China, #17005042) enriched with 50 mg/mL of bovine pituitary extract (Corning, Shanghai, China, #354123), 5 ng/mL of epidermal growth factor (Corning, Shanghai, China, #354001), 100 U/mL of penicillin, and 100 U/mL of streptomycin. PCa cells were consistently maintained in phenol red-positive RPMI-1640 medium (Thermo Fisher, Shanghai, China, #11875085) supplemented with 10% fetal bovine serum (FBS, Sigma-Aldrich, Guangzhou, China, #F0193) in an incubator set at 37°C with 5% CO_2_.

The establishment of PCa cells exhibiting a stable decrease in EBF1 expression was accomplished through the method of lentiviral infection. To generate the lentivirus, HEK293T cells were seeded at 2 × 10^6^ in a 10-cm^2^ dish in Opti-MEM reduced serum medium (Thermo Fisher, Shanghai, China, #31985062) and cultured overnight to reach 70–80% confluent. Subsequently, pLenti-shEBF1 (#VB900214-7356fay) or a scrambled control (shCtrl, #VB010000-9526zpu) sourced from VectorBuilder (Guangzhou, China), in conjunction with the packaging vectors pCMV delta R8.2 and pCMV-VSV-G (#12263 and # 8454, AddGene, Beijing Zhongyuan, Beijing, China), were co-transfected into HEK293T cells using Xtreme gene 9 (Sigma-Aldrich, Guangzhou, China, #636577900) and cultured continually for another 48 h. Following the harvesting of lentiviral particles from the culture medium of the HEK293T cells, PCa cells were seeded at 0.5 × 10^6^ in a 10-cm^2^ plate in RPMI-1640 medium containing 8 μg/mL polybrene (Sigma-Aldrich, Guangzhou, China, #H9268) until reaching ~50% confluency. Subsequently, the PCa cells underwent lentivirus transfection at 37°C in a humidified incubator with 5% CO_2_ for 24 h, after which selection was performed using 2 μg/mL puromycin (Sigma-Aldrich, Guangzhou, China, #P7255) over a duration of 9 to 11 days. The production of PC-3/EBF1 cells through lentiviral infection was performed as previously outlined, with the exception that the target lentiviral plasmids were changed to pLV-hEBF1 (VectorBuilder, Guangzhou, China, #VB900172-5148qba) or pLV-vector (VectorBuilder, Guangzhou, China, #VB010000-9495wwg). To conduct a more in-depth examination of the biological impacts of ITPR1 overexpression in EBF1-deficient PCa cells, LNCaP^EBF1-/-^ or 22RV1^EBF1-/-^ cells were transiently transfected with pLV-hITPR1 (VectorBuilder, Guangzhou, China, #VB900214-7377ssj) or pLV-vector (VectorBuilder, Guangzhou, China, #VB010000-9495wwg), as described above.

### 2.4 Cell Proliferation and Colony Formation

To assess cell proliferation, PCa cells were plated at a density of 1.0 × 10^3^/well in a complete medium within 96-well plates. Following a culture period of 24, 48, and 72 h, cell viability was evaluated utilizing a 3-(4,5)-dimethylthiahiazo (-z-y1)-3,5-di-phenytetrazoliumromide (MTT) kit (Beyotime, Shanghai, China, #C0009S), in accordance with the manufacturer’s guidelines. The final absorbance was measured at 570 nm using a Synergy Neo2 Hybrid plate reader (BioTek, Shanghai, China).

To facilitate colony formation, cells were seeded at a density of 500 cells/well in a complete medium within 6-well plates. After a period of 14 days, the colonies were fixed using 4% paraformaldehyde (Beyotime, Shanghai, China, #P0099) and subsequently stained for 20 min with 0.1% crystal violet (Applygen, Beijing, China, #B1087). The colonies were then counted using a DMi8 inverted microscope (Leica, Shanghai, China).

### 2.5 Wound Healing and Transwell Invasion Assays

For wound healing, cells were inoculated into six-well plates at a density of 5 × 10^5^ and permitted to proliferate until they reached over 95% confluence. The monolayer was carefully and gradually scraped using a new 200-μL pipette tip across the center of the well. Subsequently, the plates were gently rinsed twice with PBS to eliminate any detached or deceased cells, and the wells were replenished with fresh serum-free RPMI-1640 medium. Following treatment, the same field in each well was photographed using a microscope at two distinct time points (0 and 24 h). The open wound areas at each observation point were assessed at various intervals under a microscope at 50× magnification.

In relation to cell invasion, 8.0 × 10^4^ cells were resuspended in RPMI-1640 medium and subsequently seeded into the upper chamber of Transwell inserts (Corning, #3422). The inserts were pre-coated with BD Matrigel (Corning, Shanghai, China, #356234). Subsequently, 15% FBS medium was introduced into the lower chamber. After a 24-h incubation period, the cells on the upper surface were scraped off and washed away, while the cells that migrated to the lower surface were fixed using 4% paraformaldehyde for 20 min, stained with 4′,6-diamidino-2-phenylindole (DAPI, Thermo Fisher, Shanghai, China, #D1306) in the dark for 5 min and counted using a fluorescence microscope. Wells were examined using five randomly chosen fields, which were counted under a microscope in three separate assessments, and the relative cell count was determined in relation to the sh-Ctrl.

### 2.6 Tumor Xenograft Experiment

Male Balb/c nude mice (age: 10 weeks; weight: 24.6 ± 3.4 g) were acquired from Beijing Vitalstar Biotechnology (Beijing, China; certification No.: SCXK (Beijing) 2023-0014) for the *in vivo* experiments. All procedures were approved by the Institutional Animal Care and Use Committee (IACUC) of Baoji Center Hospital (approval No. BZYL2023-20-02) and conducted in accordance with the ARRIVE guidelines, the NIH Guide for the Care and Use of Laboratory Animals, and local regulations. Humane endpoints were predefined as tumor volume exceeding 1500 mm^3^ or body weight loss > 20%. The mice, which were acclimatized to their environment for one week prior to the study, were housed in individually ventilated cages (IVCs) with sterile bedding and kept in a controlled room with relative humidity maintained at 55–70% and temperature conditions set between 22–25°C. A total of 128 mice were used (n = 5/group for xenograft volume assays; n = 9/group for tumor weight analysis at Day 36). Randomization was performed by an independent researcher using a random number table, with cages assigned to groups after acclimatization [13]. For the xenograft studies, 1.5 × 10^6^ PC-3 cells in a volume of 100 μL (combined with Matrigel (Corning, Shanghai, China, #356237) at a 1:1 v/v ratio) were implanted subcutaneously into the flank of each mouse using a 28-gauge needle. The size of the xenograft was measured with a caliper every three days and calculated using the formula V = 0.52 × length × width^2^ [14], with inter-observer variability < 5%. The xenografts were ultimately harvested after the animals were euthanized via CO_2_ inhalation (30% chamber displacement rate) followed by cervical dislocation for confirmation, on Day 36 following cell inoculation. Investigators measuring tumor size and performing euthanasia were blinded to group allocation.

### 2.7 Real-Time Quantitative Polymerase Chain Reaction (qPCR)

Total RNA was extracted and purified utilizing a RNeasy Mini Kit (Qiagen, Shanghai, China, #74104), with the concentration and purity assessed using a NanoDrop 2000 spectrophotometer (Thermo Fisher, Shanghai, China). Reverse transcription was subsequently performed with the RevertAid Reverse Transcription kit (Thermo Fisher, Shanghai, China, #K1691). Real-time qPCR was executed on an Applied Biosystems 7500 real-time PCR system (Thermo Fisher, Shanghai, China, #4406985) by employing 2× SYBR Green qPCR Master Mix from Yeasen (Thermo Fisher, Shanghai, China, #11201ES03). The cycling parameters consisted of a single cycle for 5 min at 95°C, followed by 40 cycles of 15 s at 95°C and 34 s at 60°C. Relative expression levels of target genes from three independent experiments were calculated using the 2^−ΔΔCt^ method [15], with GAPDH serving as the reference gene. The primers used in the current study were: EBF1 (NM_001290360.3), 5′-CCATGTCCTGGCAGTCTCTG-3′ and 5′-GGGAGTAGCTGCATGTTCCA-3′; GAPDH (NM_002046.7), 5′-CCGCATCTTCTTTTGCGTCG-3′ and 5′-GCCCAATACGACCAAATCCG-3′.

### 2.8 Western Blot

Total proteins were extracted from whole cells utilizing a Total Protein Extraction kit (Sangon Biotech, Shanghai, China, #C006225) supplemented with a protease inhibitor cocktail (Sangon Biotech, Shanghai, China, #786-108). The concentrations of the proteins were assessed using a Bicinchoninic Acid (BCA) protein assay kit (Beyotime, Shanghai, China, #P0012). A total of twenty-five micrograms of proteins were subjected to separation on an 8–15% sodium dodecyl sulfate-polyacrylamide gel electrophoresis (SDS–PAGE) and subsequently transferred to a polyvinylidene fluoride (PVDF) membrane (Bio-Rad, Wuhan, China, #1620174). The membranes underwent blocking with 5% non-fat milk in TBST buffer, were incubated with primary antibodies overnight at 4°C, and the protein bands were ultimately visualized through enhanced chemiluminescence detection (Cytiva, Shanghai, China, #RPN3004). The primary antibodies used in Western blot were: rabbit polyclonal anti-EBF1 (Thermo Fisher, Shanghai, China, #PA5-41632; 1:800), rabbit polyclonal anti-ITPR1 (Proteintech, Wuhan, China, #19962-1-AP; 1:1000) and rabbit polyclonal anti-GAPDH (Proteintech, Wuhan, China, #10494-1-AP; 1:2000). The secondary antibody used was goat anti-Rabbit IgG-HRP 2nd Ab (Abcam, Shanghai, China, #ab97200; 1:5000). Densitometric scanning of immunoblots was performed using the Image J software (National Institutes of Health, Bethesda, MD, USA).

### 2.9 Immunohistochemistry

PCa tissues and xenografts were fixed in 4% paraformaldehyde and subsequently embedded in paraffin. Tissue sections measuring 5 μm were then subjected to immunostaining as previously described [16]. Briefly, antigens were retrieved by boiling in Citrate Antigen Retrieval Solution (Beyotime, Shanghai, China, #P0083) for 20 min at 95°C. Following a gradual cooling to RT, the sections were incubated in a solution of 0.3% hydrogen peroxide-methanol for 15 min to further block endogenous peroxidase activity. The sections were then incubated overnight at 4°C with various primary antibodies in phosphate-buffered saline, followed by sequential incubation with a biotinylated secondary antibody and a streptavidin peroxidase complex provided by the VECTASTAIN Elite ABC HRP Kit (Vector Labs, Beijing, China, #PK-6100). Finally, peroxidases were visualized by incubating sections in 1 mg/mL DAB (3, 3′-diaminobenzidine tetrahydrochloride, Sigma-Aldrich, Guangzhou, China, #D5637)-PBS solution (pH = 7.4) containing 0.03% H_2_O_2_ (Sigma-Aldrich, Guangzhou, China, #7722-84-1) at RT for 5–10 min. Primary antibodies used in this study included rabbit polyclonal anti-EBF1 (Thermo Fisher, #PA5-41632, 1:150), rabbit polyclonal anti-ITPR1 (Proteintech, Wuhan, China, #19962-1-AP, 1:100), rabbit polyclonal anti-Ki67 (Abcam, Shanghai, China, #ab15580, 1:500), rabbit monoclonal anti-cleaved Caspase-3 (Cell Signaling, Shanghai, China, #9664; 1:200), rabbit polyclonal anti-E-cadherin (Proteintech, Wuhan, China, #20874-1-AP, 1:200) and rabbit polyclonal anti-Vimentin (Proteintech, Wuhan, China, #10366-1-AP, 1:400).

### 2.10 Chromatin Immunoprecipitation (ChIP)

Chromatin extracts were obtained from PC-3/EBF1 cells utilizing a Chromatin Extraction Kit (Abcam, Shanghai, China, #ab117152) following the guidelines provided by the manufacturer. The chromatin extracts were then incubated with anti-EBF1 (Cell Signaling, Shanghai, China, #50752) or control IgG antibodies at 4°C overnight, after which 60 mL of salmon sperm/protein A-agarose (Sigma-Aldrich, Guangzhou, China, #16-157) was added to facilitate the recovery of immunocomplexes. Subsequently, the bound protein–chromatin complexes were eluted using ChIP Elution Buffer (Abcam, Shanghai, China, #ab205698) at 25°C for a duration of 30 min. The isolated DNA was ultimately analyzed by PCR using primers specific to the *ITPR1* (NC_000003.12) promoter: Site 1, 5′-GATTTCTGAAATTCTTGGCT-3′ and 5′-CAGGCTGCTGAGCCTGTTCCTT-3′; Site 2, 5′-AGTTTCATGAGACTCGAT-3′ and 5′-CTGGACTCCCGTTCCAGACT-3′. In addition, we employed Histone H3 antibody (Cell Signaling, Shanghai, China, #2650)-bound *GAPDH* promoter regions as a positive control for ChIP-qPCR assays. The primers for amplifying the *GAPDH* promoter were 5′-CAGCAACAGCCCATCACCAT-3′ and 5′-GGCAGTGAGGGTCTCTCTCG-3′.

### 2.11 Nonradioactive Electrophoretic Mobility Shift Assay (EMSA)

Chromatin extracts were obtained from PC-3/EBF1 cells utilizing the Nuclear Extraction Kit (Abcam, Shanghai, China, #ab113474), following the guidelines provided by the manufacturer. To examine the direct interaction of EBF1 with the *ITPR1* promoter *in vitro*, approximately 1 μg of EBF1-enriched nuclear extracts was combined with 30 mM custom digoxigenin (DIG)-labeled probes (Sangon Biotech, Shanghai, China) that correspond to the potential EBF1 binding motifs (ATGGCGGTGGATTTCCCCAAAGACAAG) in a 20 μL reaction mixture containing 100 mM HEPES (4-(2-Hydroxyethyl)piperazine-1-ethanesulfonic acid), 5 mM EDTA (ethylenediaminetetraacetic acid, pH = 8.0), 50 mM (NH_4_)_2_SO_4_, 5 mM DTT (dithiothreitol), 1% (v/v) Tween 20, and 150 mM KCl, incubated at 30°C for 20 min. After conducting gel electrophoresis on a 6% native PAGE gel and transferring to a nylon membrane (Thermo Fisher, Shanghai, China, #77015), the protein-bound DIG-labeled probes were detected immunologically using the DIG Luminescent Detection Kit (Sigma-Aldrich, Guangzhou, China, #11363514910). Competitive inhibition assays were carried out with a 50-fold molar excess of unlabeled/cold probe or with a mutated probe.

### 2.12 Reporter Assay

The genomic fragments of the human *ITPR1* promoter, located approximately 1.8 kb upstream of the translation start site, were produced through PCR amplification utilizing human Genomic DNA (Promega, Beijing, China, #G1521) as the template. The mutation of the potential EBF1-binding site on the *ITPR1* promoter was executed (replacing “TTCCCCAAAGA” with “TGagCtTAgcT”: Lowercase letters represent mutated bases), using the QuikChange Site-Directed Mutagenesis Kit (Agilent, Beijing, China, #200513). The resulting fragments were then inserted into the SstI/NheI sites of pGL3-Basic (AddGene, Beijing Zhongyuan, Beijing, China, #212936) with the assistance of the CloneJET PCR Cloning Kit (Thermo Fisher, Shanghai, China, #K1231). For the reporter assay, 0.5 μg of the wild-type or mutant reporter plasmids and 0.02 μg pRL-TK Renilla reporter plasmids (Promega, Beijing, China, #E2231) were cotransfected into EBF1-null 293T cells (Applied Biological Materials, #T3005391), along with pCMV3-hEBF1 (Sino Biological, Beijing, China, #HG12604-UT) or empty vector (Sino Biological, Beijing, China, #CV011), using Xtreme gene 9. After 48 h, the cells were collected, and luciferase activities were measured using a dual luciferase reporter assay system (Promega, Beijing, China, #E1980).

### 2.13 Statistical Analysis

GraphPad Prism 8.0 software (GraphPad Software, Boston, MA, USA) was utilized for the creation of graphical figures and the execution of statistical analyses. All data are presented as means ± Standard Deviation (SD) and assumptions were checked and met for all statistical tests. The *Student’s t*-test was utilized to assess significant differences in comparisons between two groups, while one-way or two-way Analysis of Variance (ANOVA), accompanied by a Tukey post hoc test, was used for comparisons that involved more than two groups, with *p* values < 0.05 deemed statistically significant.

## 3 Results

### 3.1 Association of Decreased EBF1 Expression in Localized Prostate Cancer with Gleason Grade and Biochemical Recurrence (BCR)-Free Outcomes

**Figure 1:**
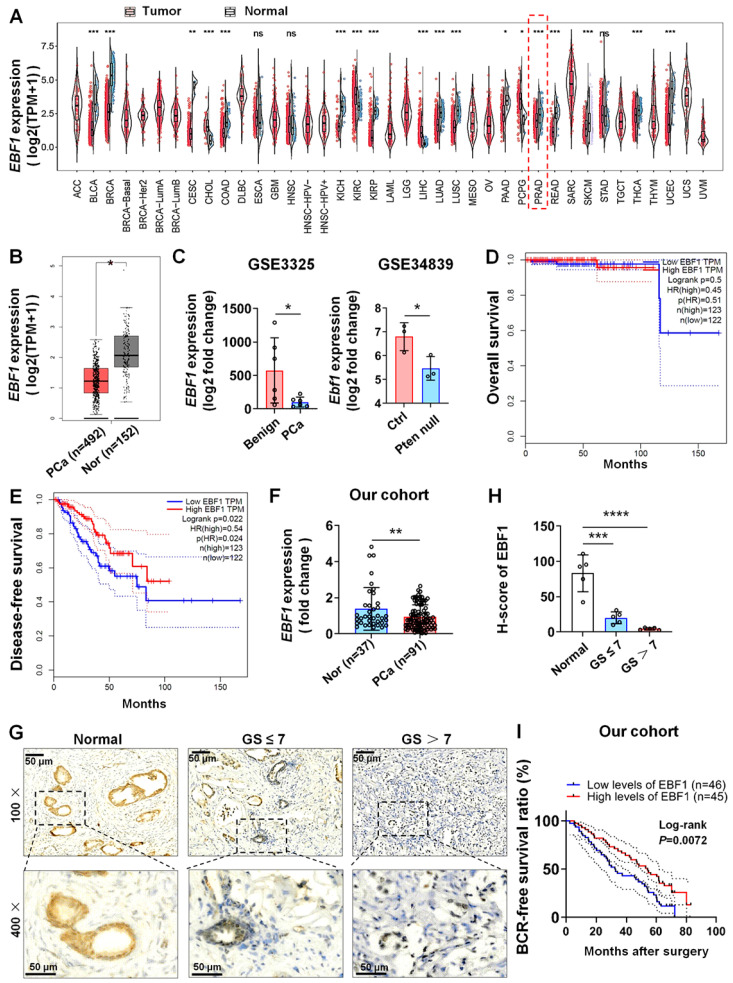
Downregulation of early B-cell factor 1 (EBF1) expression is associated with poor disease-free survival and high Gleason score in human prostate cancer (PCa). (**A**) Analysis of *EBF1* mRNA expression in different tumor and corresponding normal tissues using the TIMER2.0 database. PRAD, prostate adenocarcinoma(*p < 0.05, **p < 0.01 and ***p < 0.001; ns:not significant). (**B**) Comparison of *EBF1* mRNA expression between PCa tissues and normal prostate tissues using the Gene Expression Profiling Interactive Analysis (GEPIA) database (*p < 0.05). (**C**) *EBF1* mRNA is downregulated in PCa relative to normal prostate tissues, as well as in spontaneous prostate tumors derived from Pten-null mice compared to normal mouse prostate, evidenced by bioinformatic analysis using different Oncomine PCa datasets (*p < 0.05). (**D**) Kaplan-Meier curves of overall survival in Cancer Genome Atlas (TCGA) PCa patients with *EBF1* high or low expression divided by the Quartile. Data are from the GEPIA database. (**E**) Kaplan-Meier curves of disease-free survival in TCGA PCa patients with *EBF1* high or low expression divided by the Quartile. Data are from the GEPIA database. (**F**) Quantitative polymerase chain reaction (qPCR) analysis of *EBF1* mRNA expression in our cohort (n = 37 for normal tissues and n = 91 for PCa biopsies, two-tailed unpaired *Student’s t*-test, **p < 0.01). (**G**) Immunohistochemistry (IHC) staining of EBF1 protein in PCa and normal prostate tissues from our cohort. GS, Gleason score. Bar = 50 μm (**H**) H-scores for normal (n = 5), PCa tissues with Gleason Score (GS) ≤ 7 (n = 5) and PCa tissues with GS > 7 (n = 5) were calculated for panel (**G**) (one-way ANOVA followed by Tukey’s post-hoc test, ***p < 0.001 and ****p < 0.0001). (**I**) Kaplan-Meier curves of biochemical recurrence-free (BRF)-survival in our PCa patients with *EBF1* high or low expression divided by the median (n = 91, Log-rank (Mantel-Cox) test).

Numerous studies have documented essential roles for EBF1 in solid tumor pathogenesis [7,17,18,19]. To investigate its clinical relevance in PCa, we first analyzed TIMER2.0 data (https://compbio.cn/timer2/), which revealed significant EBF1 downregulation in multiple malignancies including breast cancer, lung cancer, and PCa (Fig. 1A). Consistent with this, *EBF1* transcript levels were markedly reduced in PCa biopsies compared to normal adjacent or benign prostate tissues based on GEPIA (Fig. 1B) and GSE3325 dataset from GEO (Fig. 1C) databases. Bioinformatics interrogation of the GSE34839 dataset further validated decreased *Ebf1* expression in spontaneous prostate tumors derived from Pten-null mice versus normal mouse prostate (Fig. 1C). Critically, GEPIA analysis demonstrated that patients with high *EBF1* mRNA expression exhibited longer disease-free survival than those with low expression, with consistent trends in overall survival (Fig. 1D,E). Of note, the analysis of overall survival within the TCGA-PRAD subset should be interpreted with caution. This is due to the paucity of mortality events and the relatively short follow-up period, which contrasts with the long natural history of PCa, as clearly highlighted elsewhere [20]. To mechanistically explore EBF1 function, we performed qPCR on hormone-naïve localized PCa biopsies, confirming significantly lower *EBF1* mRNA in tumor tissues (*p* < 0.01, Fig. 1F). Immunostaining corroborated reduced EBF1 protein levels in PCa specimens (Fig. 1G,H). In our cohort of 91 patients, low EBF1 expression significantly correlated with shorter biochemical recurrence-free survival (Fig. 1I). Collectively, these findings indicate that EBF1 downregulation is associated with PCa development and progression.

### 3.2 EBF1 Depletion Potentiates PCa Cell Proliferation, Colony Formation, Migration and Invasion Capabilities In Vitro

**Figure 2:**
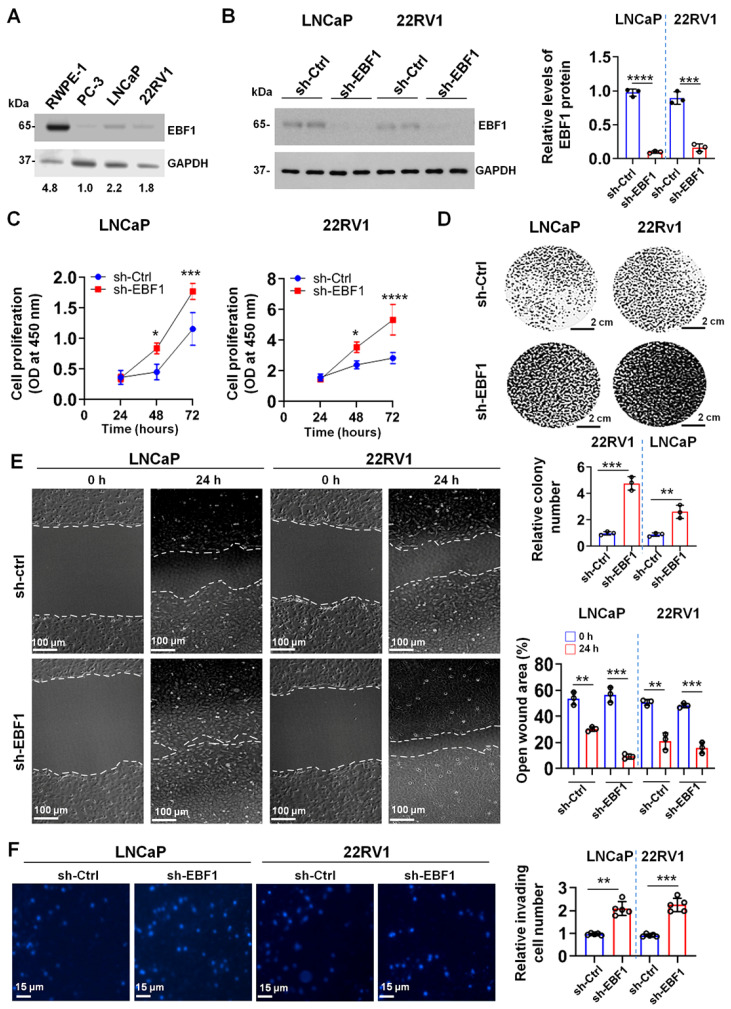
Early B-cell factor 1 (EBF1) deficiency promotes human prostate cancer cell proliferation, colony formation, migration, and invasion *in vitro*. (**A**) Western blot showing the expression of EBF1 in human prostate epithelial and prostate cancer (PCa) cell lines. (**B**) Establishment of the PCa cells that were stably deprived of endogenous EBF1 was verified by Western blot. Densitometric scanning of immunoblots was performed in which the level of a target protein was normalized against the protein level in sh-Ctrl group, which was arbitrarily set at 1 (two-tailed unpaired *Student’s t*-test, n = 3, ***p < 0.001 and ****p < 0.0001). (**C**) A 3-(4,5-Dimethylthiazol-2-yl)-2,5-diphenyltetrazolium bromide (MTT) assay was conducted to assess the proliferation of LNCaP and 22RV1 cells after EBF1 knockdown during a 3-day culture (two-way analysis of variance (ANOVA) followed with Sidak’s multiple comparisons test, n = 3, *p < 0.05, ***p < 0.001 and ****p < 0.0001). (**D**) Anchorage-dependent clonogenic ability of LNCaP and 22RV1 cells with different transfections was assessed using colony formation assay after a period of 14 days (two-tailed unpaired *Student’s t*-test, n = 3, **p < 0.01 and ***p < 0.001). Bar = 2 cm. (**E**) Representative images of wound healing in LNCaP and 22RV1 cells with different transfections at 0 and 24 h following scratch. The open wound areas were assessed under a microscope at 50 × magnification (two-tailed unpaired *Student’s t*-test, n = 3, **p < 0.01 and ***p < 0.001, bar = 100 μm). (**F**) Effects of EBF1 inhibition on cell invasiveness were evaluated using a Transwell assay, after a 24-h incubation period (n = 5, **p < 0.01 and ***p < 0.001, bar = 15 μm). Relative invading cell number quantifies Matrigel-invaded cells normalized to the wild-type (WT) control group #1 (set as 1.0). we used Welch’s unequal variances *t*-test (not assuming equal variance) to compare sh-EBF1 versus sh-Ctrl, with F-test confirming variance heterogeneity (LNCaP: sh-EBF1 versus sh-Ctrl, F = 48.04, *p* = 0.0025; 22RV1: sh-EBF1 versus sh-Ctrl, F = 27.43, *p* = 0.0072).

To define EBF1’s functional role in PCa, we conducted a series of *in vitro* assays. Western blot analysis detected EBF1 expression across all tested cell lines, with the highest level in RWPE-1, intermediate in 22RV1/LNCaP, and lowest in PC-3 cells (Fig. 2A). Based on this gradient, we selected LNCaP and 22RV1 for knockdown experiments. Lentiviral delivery of shEBF1 followed by puromycin selection achieved efficient EBF1 knockdown in LNCaP and 22RV1 cells (Fig. 2B). Compared to scrambled shRNA controls, EBF1-knockdown cells exhibited higher proliferation over a three-day culture period (Fig. 2C), increased efficiency in colony formation after 14 days (Fig. 2D) and significant enhancement of migration/invasion capacity (Fig. 2E,F). These results indicate that EBF1 depletion enhances PCa cell aggressiveness, supporting its potential tumor-suppressive function.

### 3.3 Ectopic Overexpression of EBF1 Sppressess Prostate Cancer Growth In Vivo

**Figure 3:**
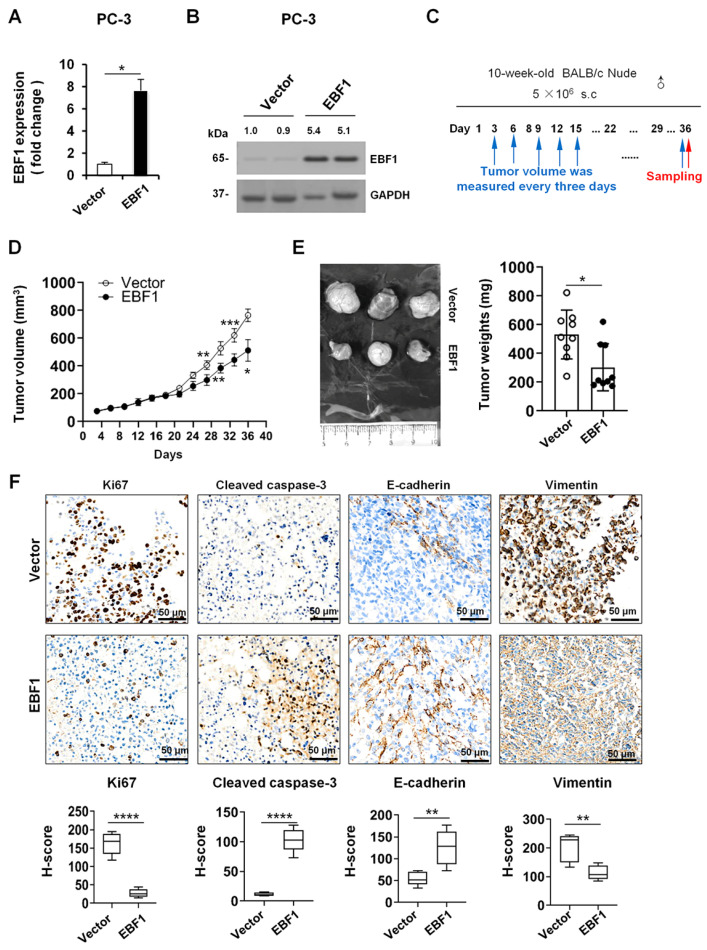
Repression of PC-3 prostate cancer (PCa) cell growth *in vivo* via ectopic overexpression of early B-cell factor 1 (EBF1). (**A**) Generation of the PC-3/EBF1 cells was validated by quantitative polymerase chain reaction (qPCR) analysis (two-tailed unpaired *Student’s t*-test, n = 3, *p < 0.05). (**B**) Generation of the PC-3/EBF1 cells was confirmed at the protein level using Western blot. (**C**) Scheme showing the protocol of the xenograft model. (**D**) PC-3 cells with different transfections were injected into left flanks of male nude mice. Tumor growth was examined every three days for 36 days (two-way ANOVA followed with Sidak’s multiple comparisons test, n = 5, *p < 0.05, **p < 0.01 and ***p < 0.001). (**E**) Comparison of tumor weights at d 36 after cell inoculation (two-tailed unpaired *Student’s t*-test, n = 9, *p < 0.05). (**F**) Ki67, Cleaved caspase-3, E-cadherin and Vimentin expressions were examined in xenografts using immunohistochemistry and corresponding H-scores of different biomarkers were calculated accordingly (two-tailed unpaired *Student’s t*-test, n = 5, **p < 0.01 and ****p < 0.0001, bar = 50 μm).

To validate the tumor-suppressive role of EBF1 *in vivo* suggested by loss-of-function studies, we stably overexpressed EBF1 in PC-3 cells (minimal endogenous expression). Lentiviral transfection achieved an approximate 6.7-fold increase in *EBF1* mRNA expression levels and a roughly 5.2-fold elevation in EBF1 protein levels, as illustrated by qPCR (Fig. 3A) and Western blot (Fig. 3B) analyses, respectively. Subsequently, 1.5 × 10^6^ vector-control or EBF1-overexpressing cells were injected subcutaneously into flanks of BALB/c nude mice (n = 5/group; Fig. 3C). Tumor growth monitoring revealed that from 28 day, EBF1-overexpressing tumors showed 58% smaller volume vs. controls (Fig. 3D). At day 36, EBF1-overexpressing tumors weighed 63% less (304.78 ± 164.52 mg vs. 531.33 ± 169.79 mg; *p < 0.05, Fig. 3E). Following immunostaining of tumorigenic tissues, significant distinctions between EBF1 wild-type tumors and those overexpressing EBF1 were further unmasked. Specifically, xenografts derived from EBF1-overexpressing cells exhibited reduced levels of the cell proliferation marker Ki67 (****p < 0.0001), while showing increased levels of the cell apoptosis marker Cleaved caspase-3 (****p < 0.0001). Importantly, this tumorigenesis-inhibiting characteristic ultimately resulted in a decrease in tumor aggressiveness, as EBF1-overexpressing tumors demonstrated elevated E-cadherin expression (**p < 0.01) alongside a reduction in Vimentin expression (**p < 0.01), both of which are canonical markers associated with the epithelial-mesenchymal transition (EMT) (Fig. 3F). These data collectively indicate that EBF1 overexpression suppresses PCa tumorigenicity *in vivo* through inhibiting proliferation, promoting apoptosis, and attenuating EMT. Of note, the experimental strategies used *in vitro* and *in vivo* are not fully aligned in our study. Specifically, shRNA-mediated knockdown in LNCaP and 22RV1 cells is used *in vitro*, whereas EBF1 overexpression in PC-3 cells is employed *in vivo*. The reasoning behind the differences in experimental design (Knockdown *in vitro* versus Overexpression *in vivo*) primarily arises from the distinct focus and objectives of each model system. On one hand, the primary goal of *in vitro* study here is mechanistic exploration and rapid phenotype screening. Using shRNA-mediated knockdown is efficient and well-established for quickly assessing the loss-of-function consequences of a gene in a controlled environment. It allows for direct observation of how EBF1 deficiency affects fundamental PCa cell behaviors like proliferation, migration, and invasion in specific cell lines. This approach is ideal for identifying if EBF1 loss promotes malignancy and for initial downstream target identification (like ITPR1). On the other hand, the primary goal of our *in vivo* study shifts towards therapeutic potential and relevance within a complex biological system. Overexpressing EBF1 mimics a potential therapeutic strategy (e.g., gene therapy or pharmacological activation). PC-3 cells are known for their high metastatic potential and androgen independence, making them suitable for assessing tumor growth and metastasis *in vivo*. Using overexpression allows us to test whether restoring EBF1 function can inhibit tumorigenesis in a setting that includes interactions with the tumor microenvironment (TME), immune cells, blood vessels, and extracellular matrix-factors absent *in vitro*. Therefore, the differing experimental strategies and endpoints are largely justified by the distinct purposes and inherent strengths/limitations of *in vitro* versus *in vivo* models, coupled with practical considerations of resource allocation. Employing both knockdown and overexpression strategies, albeit in different systems, provides complementary evidence strengthening the core hypothesis that EBF1 acts as a tumor suppressor in PCa.

### 3.4 Identification of ITPR1 (Inositol 1,4,5-Trisphosphate Receptor Type 1) As the Transactivation Target of EBF1 in PCa Cells

**Figure 4:**
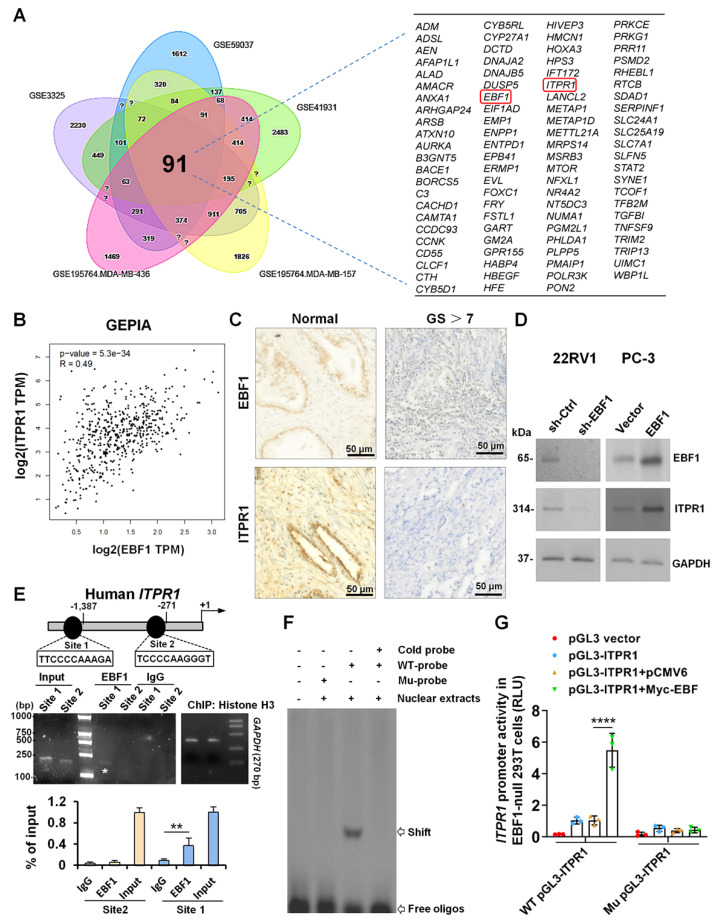
Transcriptional regulation of inositol 1,4,5-trisphosphate receptor type 1 (*ITPR1*) by early B-cell factor 1 (EBF1) in prostate cancer (PCa) cells. (**A**) The Venn diagram showing Differentially Expressed Genes (DEGs) from five datasets identifies 91 common genes that may be downstream targets of the EBF1 signaling. (**B**) The Gene Expression Profiling Interactive Analysis (GEPIA) database displayed a positive correlation between *ITPR1* mRNA levels and *EBF1* mRNA levels in prostate adenocarcinoma (PRAD). (**C**) IHC staining of EBF1 and ITPR1protein in PCa and normal prostate tissues from our cohort. Bar = 50 μm (**D**) Western blot analysis of EBF1 and ITPR1protein expression in the PCa cells with different transfections. (**E**) Chromatin immunoprecipitation-quantitative polymerase chain reaction (ChIP-qPCR) analysis in the PC-3/EBF1 cells demonstrated the recruitment of EBF1 onto a specific region of the human *ITPR1* promoter (one-way analysis of variance (ANOVA) followed by Tukey’s post-hoc test, n = 3, *p < 0.05 and **p < 0.01). Additionally, Histone H3 antibody-bound *GAPDH* promoter regions were used as a positive control. (**F**) Nuclear protein extracts were prepared from the PC-3/EBF1 cells and subsequent EMSA was conducted using a DIG-labeled probe containing the consensus EBF1 binding sequence. A competition assay was performed either a 50-fold molar excess of unlabeled/cold probe or with a mutated probe (n = 2). (**G**) The wild-type or mutated pGL3-Luc-hITPR1 reporter plasmid and pRL-TK Renilla reporter plasmid were cotransfected into EBF1-null 293T cells, along with pCMV3-hEBF1 or empty vectors as indicated. Forty-eight hours later, cells were harvested and subjected to measurement of luciferase activities using a Promega dual luciferase reporter assay kit (n = 3, two-way ANOVA followed by Tukey’s post-hoc test), ****p < 0.0001.

To elucidate the molecular mechanisms underlying EBF1 function, we analyzed public microarray datasets from the Gene Expression Omnibus (GEO). We utilized four datasets: GSE3325 (benign vs. primary PCa tissues), GSE59037 (shCtrl vs. shEbf1in colorectal cells), GSE41931 (WT vs. Ebf1^-/-^ bone marrow), and GSE195764 (shCtrl vs. shEbf1 in breast cancer cells). Using GEO2R and Venn analysis, we identified 91 overlapping differentially expressed genes (DEGs; Fig. 4A). We then screened these DEGs against three criteria: (1) Expression correlation with EBF1 (GEPIA). (2) Functional consistency (tumor suppressor if positively correlated), and *vice versa*. (3) EBF1 binding site (predicted by JASPAR *de novo* motif analysis). Specifically, ITPR1 emerged as a top candidate: ITPR1 and EBF1 showed strong co-expression in PCa and normal prostate tissues (Spearman *r* = 0.49, *p* < 0.0001, Fig. 4B). Given protein levels better reflect functional impacts, immunohistochemistry of clinical specimens confirmed reduced.

ITPR1 in EBF1-deficient contexts (Fig. 4C). *In vitro*, EBF1-knockdown 22RV1 cells exhibited significantly lower ITPR1 protein, while EBF1-overexpressing PC-3 cells showed higher levels (Fig. 4D). JASPAR analysis revealed two putative EBF1 binding sites in the *ITPR1* promoter. Subsequent ChIP assays using primers to the proximal site (Site 1) confirmed *in vivo* binding in PC-3/EBF1 cells, with a clear band in anti-EBF1 samples vs. IgG controls (Fig. 4E). EMSA using nuclear extracts from PC-3/EBF1 cells further validated direct binding *in vitro*, showing shifted bands with DIG-labeled probes that were competed by cold probes (50-fold excess) but not mutants (Fig. 4F). Consistently in luciferase reporter assays, the wild-type or mutated pGL3-Luc-hITPR1 reporter plasmid (replacing “TTCCCCAAAGA” with “TGagCtTAgcT”) and pRL-TK Renilla reporter plasmid, along with pCMV3-hEBF1 or empty vectors, were cotransfected into EBF1-null 293T cells. 48 h later, cells were harvested and dual luciferase reporter assay showed that EBF1 overexpression increased *hITPR1* promoter activity by ~4.3-fold. Site-directed mutation of the binding sites abolished this induction (Fig. 4G and Fig. S1). Collectively, these results demonstrate that EBF1 directly regulates *ITPR1* transcription in PCa cells.

### 3.5 Therapeutic Effects of ITPR1 Overexpression in Mitigating the Malignant Behavior Exacerbated by EBF1 Depletion in PCa Cells

**Figure 5:**
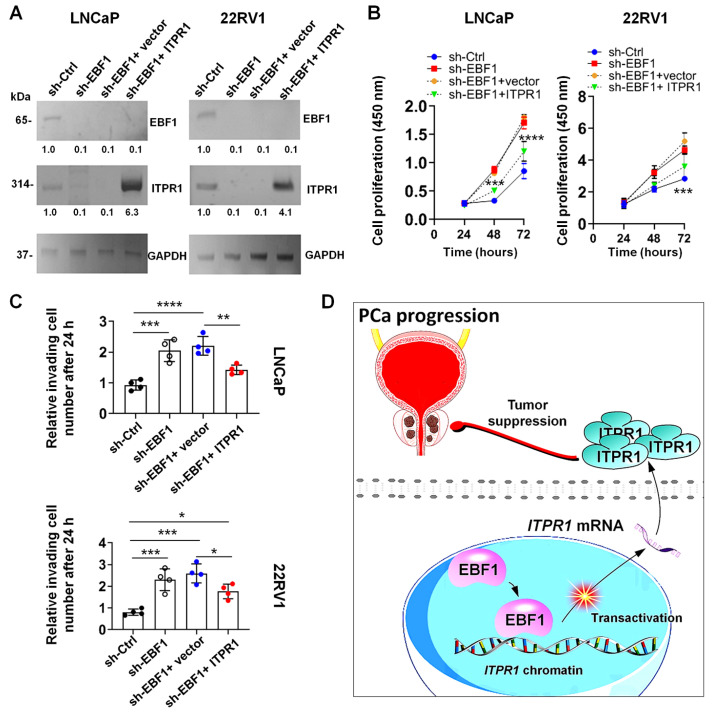
Ectopic expression of inositol 1,4,5-trisphosphate receptor type 1 (*ITPR1*) ameliorates the early B-cell factor 1 (EBF1) deficiency-potentiated malignant behaviors in PCa cells. (**A**) LNCaP^EBF1-/-^ or 22RV1^EBF1-/-^ cells were transiently infected with pLV-hITPR1 or an empty lentiviral vector. 48 h later, cells were harvested and subjected to Western blot analysis. (**B**) A 3-(4,5-Dimethylthiazol-2-yl)-2,5-diphenyltetrazolium bromide (MTT) assay was conducted to assess the proliferation of LNCaP^EBF1-/-^ or 22RV1^EBF1-/-^ cells following ITPR1 overexpression during a 3-day culture (two-way analysis of variance (ANOVA) followed with Sidak’s multiple comparisons test, n = 3, ***p < 0.001 and ****p < 0.0001). (**C**) Effects of ITPR1 overexpression on cell invasiveness in LNCaP^EBF1-/-^ or 22RV1^EBF1-/-^ cells were evaluated using a Transwell assay, after a 24-h incubation period (two-tailed unpaired *Student’s t*-test, n = 4, *p < 0.05, **p < 0.01, ***p < 0.001 and ****p < 0.0001). (**D**) Proposed working model for the current study: EBF1 serves as a crucial tumor suppressor in PCa. The absence of EBF1 significantly influences the increased aggressiveness of PCa cells, especially in enhancing their proliferation, migration, clonogenicity, and *in vivo* tumorigenic potential. A fundamental mechanism underlying EBF1’s function is the transcriptional regulation of the *ITPR1* signaling pathway.

The aforementioned data indicate that EBF1 could regulate ITPR1 signaling in PCa cells by directly activating its transcription. To functionally characterize this relationship, we transiently transfected LNCaP^EBF1-/-^ or 22RV1^EBF1-/-^ cells with pLV-hITPR1 or an empty lentiviral vector. After 48 h, Western blot analysis revealed that transient transfection with pLV-hITPR1 significantly increased ITPR1 expression levels in EBF1-deficient PCa cells, but did not restore EBF1 expression. This result supports the notion that ITPR1 likely functions downstream of the EBF1 signaling pathway (Fig. 5A). In accordance with this proposition, the increase in cell proliferation (Fig. 5B) and cell invasion (Fig. 5C) resulting from EBF1 depletion was significantly reversed by ITPR1 overexpression in the LNCaP^EBF1-/-^ or 22RV1^EBF1-/-^ cells. Together, the available data strongly suggest that *ITPR1* serves as a direct transcriptional target and key downstream effector of EBF1 in PCa cells.

## 4 Discussion

Although localized prostate cancer (PCa) demonstrates favorable long-term survival, advanced disease remains incurable despite multimodal therapeutic approaches. Mortality is predominantly driven by tumor progression that escapes hormonal control [21]. In this study, we observed consistent downregulation of EBF1 in PCa cells irrespective of AR status, with expression levels progressively decreasing with increasing malignancy grade (e.g., PC-3 vs. LNCaP). These findings support the hypothesis that EBF1 loss may contribute to the acquisition of aggressive traits in early-stage PCa. Notably, while our *in vitro* data reveal no statistically significant associations between EBF1 and AR pathway, these findings do not establish causality or direct interaction. The relationship remains hypothesis-generating, pending functional interrogation via androgen/AR modulation experiments to conclusively exclude causal interactions and define their context-dependent roles in PCa progression.

EBF1 dysregulation was initially implicated in B-lineage acute lymphoblastic leukemia pathogenesis, recurrence, and therapeutic resistance. As a pivotal transcription factor, it engages chromatin to facilitate accessibility via BRG1 chromatin remodeler recruitment, thereby stabilizing B-lineage transcriptional programs [22]. Recent evidence reveals EBF1’s context-dependent roles in solid tumors: overexpressed in breast, bladder, and thyroid carcinomas yet suppressed in gastric and cervical malignancies [7]. Notably, its dual oncogenic/tumor-suppressive functions occur even within single tumor types (e.g., colorectal cancer) [23,24]. This functional dichotomy may stem from: (1) EBF1’s adaptability to tumor-specific microenvironments, and (2) divergent biological activities of downstream targets. Clinical focus on EBF1 signaling networks has intensified over two decades, exemplified by its HIF1α-p300 interaction that attenuates hypoxia-response pathways in breast cancer [25], versus AKR1B1 suppression inhibiting gastric cancer progression [26]. Collectively, EBF1 emerges as a functionally ambivalent factor capable of modulating carcinogenic pathways bidirectionally. Our Oncomine analysis confirmed significant EBF1 mRNA reduction in PCa versus normal prostates. Functional assays demonstrated that EBF1 knockdown enhanced PCa cell proliferation, migration, and invasion *in vitro*, while its overexpression inhibited tumor growth and invasiveness in murine xenografts. These consistent findings across cell models, combined with prior evidence [11], collectively establish EBF1 as a pivotal tumor suppressor in prostate carcinogenesis.

ITPR1 modulates intracellular calcium flux to orchestrate pivotal physiological processes including signal transduction, apoptosis, and proliferation. Its dysregulation perturbs cellular homeostasis, contributing to oncogenic transformations [27]. Clinically, ITPR1 downregulation correlates with PCa progression and may serve as a diagnostic biomarker [28]. Post-translational modifications dynamically regulate ITPR1 stability and subcellular localization, fine-tuning its functional outputs [29]. Mechanistically, our biochemical experiments provide novel evidence for the hypothesis that the *ITPR1* gene may employ a form of transcriptional regulation (such as by EBF1) to adjust its activation and intensity in response to stimulations mediated by paracrine signaling in PCa cells. This hypothesis is substantiated by the identification of an evolutionarily conserved EBF1-binding site at positions −1387 to −1397 within the *ITPR1* promoter-distinct from previously characterized regulatory loci, specifically from −1426 to −1435 or from −1847 to −1856 bp for hairy and enhancer of split 1 (HES1) [30], at −174 bp for estrogen receptor beta 1 (ERβ1) [31], from −101 to −89 bp for phospholipid scramblase 1 (PLSCR1) [32], at −52 bp for nuclear factor kappa-B (NF-κB) [33], and at −747 bp for the steroid hormone response element (HRE) [34]. Moreover, CAATT enhancer-binding protein element (C/EBP) may serve as a stimulatory transcription factor, spanning −528 to +169 bp region of the *Itpr1* gene, induces normal expression patterns of ITPR1 in transgenic mice [35]. In contrast, the promoter region of *ITPR1*, which is bound by EBF1 (−1387 to −1397 nucleotides) in our study, was not part of the previously mentioned *cis*-acting elements. These findings delineate a complex transcriptional hub governing ITPR1 expression. The EBF1-ITPR1 axis, potentially coordinated with other coactivators, represents a novel regulatory layer in PCa pathogenesis, offering mechanistic insights into calcium signaling dysregulation during malignant progression.

Transcriptomic profiling across diverse cancer lineages reveals that EBF1 overexpression downregulates multiple malignancy-promoting pathways, mediated through EBF1-dependent signaling [36,37]. Intriguingly, all identified pathways show suppressed activity upon EBF1 elevation [19,38,39,40]. This supports EBF1 as a broad-spectrum regulator capable of inhibiting oncogenic signaling cascades. Our *in vivo* data demonstrate that ectopic EBF1 expression in PC-3 cells reduces clonogenic capacity and diminishes invasiveness in xenograft tumors. Despite these effects, current clinical modalities lack feasible strategies for targeted gene overexpression in tumors [41,42,43,44]. Consequently, our findings necessitate alternative therapeutic approaches. The mechanisms governing EBF1 expression remain incompletely characterized; future research may reveal clinically actionable targets. For example, heterogeneous nuclear ribonucleoprotein C (HNRNPC) stabilizes *EBF1* mRNA to modulate protein abundance [45]. Alternatively, modulating EBF1 activity rather than expression offers promise. Approximately fifty percent of the EBF1 protein comprises a regulatory domain [46], where posttranslational modifications (PTMs) such as phosphorylation [47] and ubiquitination [48] occur under stress conditions. These PTM-derived isoforms, critically relevant to EBF1 function, provide precise targets for mechanistic studies and drug development. Small molecules targeting these isoforms can be optimized for enhanced ligand efficiency, potency, and selectivity [49], advancing therapeutic innovation against cancer progression.

This study has several limitations: (i) Incomplete mechanistic elucidation: The study focused on ITPR1 as a key downstream effector but did not comprehensively address other potential EBF1-regulated pathways (e.g., calcium-independent mechanisms) that may contribute to PCa progression. In addition, the interplay between EBF1 and AR signaling was not experimentally validated, leaving open questions about context-dependent interactions in hormone-sensitive versus castration-resistant PCa. (ii) Unresolved contextual duality of EBF1: While EBF1 exhibited tumor-suppressive effects in this study, its reported oncogenic roles in other cancers (e.g., breast carcinoma) suggest tissue-specific functionality. The study did not dissect the molecular determinants (e.g., cofactors, epigenetic context) underlying this duality. (iii) Clinical translation challenges: Despite robust evidence for EBF1’s tumor-suppressive role, direct therapeutic targeting of transcription factors remains clinically impractical. The study did not explore pharmacological strategies to modulate EBF1 activity (e.g., small-molecule stabilizers or PTM-targeted agents), which limits immediate translational relevance. Addressing these limitations could involve multi-omics profiling of EBF1 networks, development of EBF1-targeting therapeutics, and validation in patient-derived organoids or genetically engineered mouse models.

## 5 Conclusions

In summary, EBF1 deficiency promotes aggressive phenotypes in PCa cells, characterized by enhanced proliferation, migration, clonogenic potential, and *in vivo* tumorigenesis. Mechanistically, we demonstrate that EBF1 knockdown directly suppresses *ITPR1* transcriptional activation (Fig. 5D), revealing its tumor-suppressive function through transcriptional regulation of the ITPR1 signaling pathway. These results deepen our mechanistic insight into EBF1-mediated transcriptional control during PCa development and could enable patient stratification for potential EBF1-directed therapeutic interventions. Clinically, high EBF1 expression is significantly associated with extended BCR-free survival, underscoring its utility as a prognostic biomarker in PCa management.

## Data Availability

The authors confirm that the data supporting the findings of this study are available within the article and its Supplementary Materials.

## Supplementary Materials

The supplementary material is available online at https://www.techscience.com/doi/10.32604/or.2026.078850/s1.
