# Supplementary material for: EBF1 Deficiency Drives Prostate Cancer Progression by Interfering with the Transcriptional Regulation of ITPR1
Source: Oncol Res. 2026 Jun 16;34(7):22. doi: 10.32604/or.2026.078850 (PMC13291987; doi:10.32604/or.2026.078850)
Supplement: Supplementary file 1 [file OncolRes-34-78850-s001.zip › Fig._S1.docx]

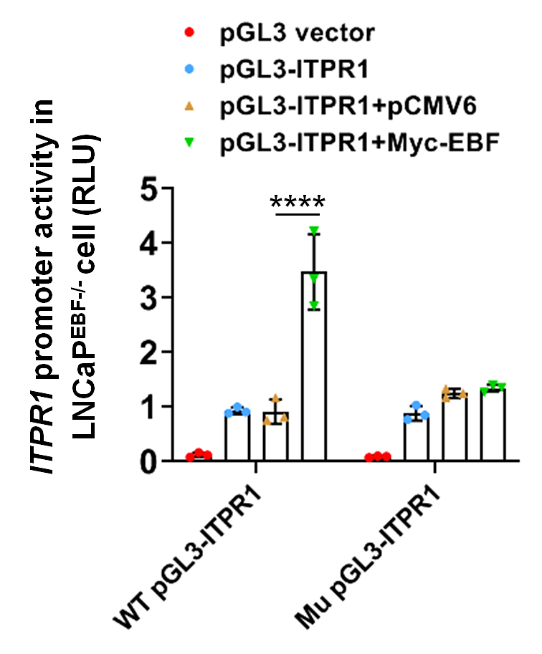


**Fig. S1** The wild-type or mutated pGL3-Luciferase-human ITPR1 (pGL3-Luc-hITPR1) reporter plasmid and renilla luciferase-thymidine kinase (pRL-TK) reporter plasmid were cotransfected into LNCaP^EBF-/-^ cells, along with pCMV3-hEBF1 or empty vectors as indicated. Forty-eight hours later, cells were harvested and subjected to measurement of luciferase activities using a Promega dual luciferase reporter assay kit (n=3, two-way ANOVA followed by Tukey’s post-hoc test), *****P* < 0.0001.
