# Supplementary material for: EBF1 Deficiency Drives Prostate Cancer Progression by Interfering with the Transcriptional Regulation of ITPR1
Source: Oncol Res. 2026 Jun 16;34(7):22. doi: 10.32604/or.2026.078850 (PMC13291987; doi:10.32604/or.2026.078850)
Supplement: Supplementary file 1 [file OncolRes-34-78850-s001.zip › Table_S1.docx]

**Supplementary Table S1** General characteristics of clinical PCa patients at diagnosis.

| **Variables** | **Benign prostatic tissues**  **(n=37)** | **Hormone naïve clinically localized PCa (n=91)** | ***P*-value** |
| --- | --- | --- | --- |
| **Median age (yrs) (range)** | 65.4 (46-74) | 69.3 (53-78) | 0.0037^a^ |
| **PSA (ng/ml)** | 1.8±0.8 | 10.8±4.9 | <0.0001 ^a^ |
| **pTNM** |  |  | >0.9999 ^b^ |
| pT2 | - | 62 |  |
| pT3 | - | 29 |  |
| **Gleason score (%)** |  |  | >0.9999 ^b^ |
| ≤ 7 | - | 67 |  |
| 8-10 | - | 24 |  |

**^a^**Two-tailed unpaired *Student's t*-test; **^b^**Fisher's exact test. Statistical significance was established as *P*<0.05.
